# XFinger-Net: Pixel-Wise Segmentation Method for Partially Defective Fingerprint Based on Attention Gates and U-Net

**DOI:** 10.3390/s20164473

**Published:** 2020-08-10

**Authors:** Guo Chun Wan, Meng Meng Li, He Xu, Wen Hao Kang, Jin Wen Rui, Mei Song Tong

**Affiliations:** 1 Department of Electronic Science and Technology, Tongji University, Shanghai 200092, China; wanguochun@tongji.edu.cn (G.C.W.); 1832943@tongji.edu.cn (M.M.L.); xuhe93@126.com (H.X.); 1830770@tongji.edu.cn (W.H.K.); 2 Sino-German College of Applied Science, Tongji University, Shanghai 200092, China

**Keywords:** deep learning, fingerprint segmentation, partial defect, U-Net, attention gates

## Abstract

Partially defective fingerprint image (PDFI) with poor performance poses challenges to the automated fingerprint identification system (AFIS). To improve the quality and the performance rate of PDFI, it is essential to use accurate segmentation. Currently, most fingerprint image segmentations use methods with ridge orientation, ridge frequency, coherence, variance, local gradient, etc. This paper proposes a method of XFinger-Net for segmenting PDFIs. Based on U-Net, XFinger-Net inherits its characteristics. The attention gate with fewer parameters is used to replace the cascaded network, which can suppress uncorrelated regions of PDFIs. Moreover, the XFinger-Net implements a pixel-level segmentation and takes non-blocking fingerprint images as an input to preserve the global characteristics of PDFIs. The XFinger-Net can achieve a very good segmentation effect as demonstrated in the self-made fingerprint segmentation test.

## 1. Introduction

The purpose of fingerprint image segmentation is to separate the fingerprint foreground from the fingerprint image, which is one of the determinants of performance in the automatic fingerprint recognition system. Even though the Automated Fingerprint Identification System (AFIS) is effective in matching a test sample fingerprint image with an already stored fingerprint image in the database, partial or latent fingerprint image still suffers from low performance rate, posing a great challenge to AFIS. The fingerprint image segmentation separates the effective region, including the ridge line and the valley line, from the fingerprint image to be segmented, thereby achieving the standard of fingerprint recognition, reducing the computational complexity, and thus improving the processing speed of the fingerprint recognition system. Despite the fact that the existing fingerprint image segmentation algorithm is able to separate the foreground region from the background region for the normal full fingerprint, it cannot function well for low-quality fingerprint images with partial distortion in the foreground region. Online fingerprint recognition is becoming increasingly common in daily life scenes, but not every fingerprint press can get complete and clear fingerprints. Fingerprint images with partial defects often appear in identity authentication due to damage to the fingerprint surface, contamination of the fingerprint surface, or damage or contamination of the fingerprint sensor. Research in fingerprint segmentation has been making low progress while that in image processing and computer vision is developing more and more rapidly. Deep learning and Convolutional Neural Networks (CNN) continue to advance their research in computer vision. In terms of fingerprint segmentation research, the traditional algorithm has always been used for the segmentation of fingerprints. The traditional algorithm is mainly divided into two categories, pixel-wise and block-wise. The latter is more frequently adopted. In the block-wise method, the fingerprint images are usually divided into different non-overlapping blocks of equal size according to different block-wise features of the fingerprints, and then the blocks are further classified into foreground and background regions. The most commonly used features in segmentation algorithms include grayscale features, directional features, ridge and ridge frequency features, ridge strength features [1], and frequency domain features [2]. The traditional algorithm has high requirements on the quality of the image, and the segmentation result obtained by the partial or the defective image is not ideal. The fingerprint segmentation in fingerprint identification using the traditional segmentation algorithm appears to be poorly robust and cannot accurately segment the local defect fingerprints studied in this paper.

The fingerprint segmentation method of XFinger-Net proposed in this paper can solve the above problems. Based on U-Net, it inherits U-Net’s characteristics of requiring very few annotation images and reasonably short training time due to data enhancement and elastic deformation. The attention gate with fewer parameters used in the XFinger-Net replaces the cascaded network, aiming to suppress uncorrelated regions in the input image while highlighting the region of interest (RoI) of the partially defective fingerprint images (PDFIs), forming an important part of this study. Attention gate is not a superposition of simple models but a weight mask generator for each single network branch. The incremental nature of the superimposed network structure can gradually refine the attention to complex images. In other words, after passing through multiple attention gates, the network already knows the location of the region of interest and gives high weight. The residual mechanism is used not only in the residual unit of U-Net but also in the attention module, which suppresses gradient disappearance and explosion and feature loss. Instead of taking non-overlapping segmented fingerprint images as input for binary classification tasks, XFinger-Net implements pixel-level segmentation and takes an undivided fingerprint image as input, preserving the global characteristics of the fingerprint image. The loss function in this work is also modified thus that the bull’s-eye is the same as the Roi exported by the network. Experimental data demonstrated that XFinger-Net achieved very good performance in the fingerprint segmentation. The fingerprint image segmentation could eliminate the interference of background noise, remove the pseudo feature points in the background, reduce the false feature points brought by the defect area in the foreground of the fingerprint, and improve the fingerprint recognition rate. In addition, the use of XFinger-Net reduced the cost of preprocessing steps in AFIS. In the aspect of fingerprint image segmentation, Ratha et al. (1995) [3] proposed 16 × 16 blocks of classes, among which each one was developed based on the gray scale variance in the direction opposite to the orientation of ridges. With the intention to obtain a smooth image, Bazen and Gerez (2000) [4] used the coherence and the morphology of fingerprint image to filter different types of noises. Naji et al. (2002) [5] developed a segmentation algorithm that computerized or automated the method of selecting a threshold value at the time of segmentation with the aid of histogram equalizer. Zhu et al. (2006) [6] also used neural network concepts to train the fingerprint dataset, but they used the gradient of the fingerprint orientation to segment the images. Thai, Huckemann, 2016 [7] proposed a new approach for fingerprint segmentation from three aspects. Firstly, they used factorized directional bandpass (FDB) and directional Hilbert transform originated from Butterworth bandpass (DHBB) filter combined with soft-thresholding for texture extraction. Secondly, they manually marked 10560 images as an evaluation benchmark for ground truth segmentation. Thirdly, they compared systematically factored directional filtering with other similar fingerprint segmentation approaches and obtained better performance. In 2018, Dinh-Luan Nguyen, Kai Cao, and Anil K. Jain proposed a fingerprint image segmentation method and a deep network model SegFinNet [8] in the field of latent fingerprints. Serafim, P. B. S., Medeiros, A. G., Rego, P. A., et al., 2019 [9] proposed an ROI segmentation method based on CNN to classify image patches and apply filtering techniques for improving the quality of the ROI.

Attention-based image classification [10] and semantic-based segmentation architecture [11] have attracted widespread attention. In practical applications, some medical image tasks are solved by using attention mechanisms. For example, attention gate has been used to segment Computed Tomography (CT) images [12]. The gate model implicitly learns to suppress irrelevant regions in the input image while highlighting salient features that are useful for a particular task. In the field of face recognition, face detection uses a concatenated convolution network, and the attention model eliminates the necessity of cascading.

## 2. Methodology

### 2.1. Data and Raw Materials

The present study used a total of 800 fingerprints, of which 640 were training data and 160 were test data. After data generation, a total of 6400 fingerprint images became our training data. All of the data had the same 512 × 512 in-plane size, but the fingerprint images did not necessarily have the same resolution. The training data were divided into two categories, defective fingerprints and completive fingerprints, of which 60% were defective fingerprints and 40% were completive fingerprints. The fingerprint dataset consisted of three parts. The first part was the Fingerprint Verification Competition (FVC) dataset, which included FVC2000, FVC2002, FVC2004, and FVC2006 [13,14,15,16]. Pick out the fingerprint images suitable for the research of this topic. The second part used the LivDet [17,18,19] dataset, which accessed for images used in the Fingerprint Liveness Detection Competitions 2009, 2011, 2013, and 2015. The third part used the fingerprint image collected by ZKTeco company’s Liver20 fingerprint collector to simulate the fingerprint defect and the collector stain to obtain the fingerprint image of the online defect.

**Figure 1:**
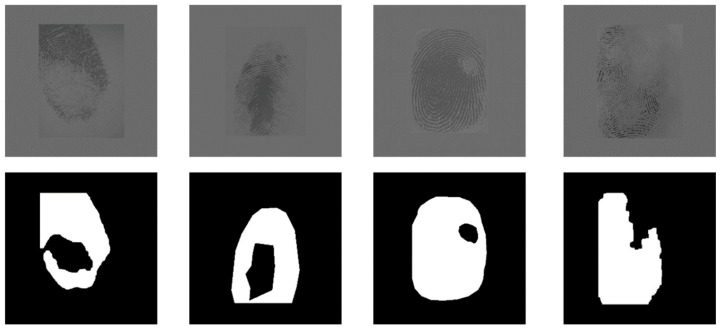
Original fingerprint and corresponding label.

The labeling of the training samples using herein was primarily based on whether the Gabor enhancement method [20] was valid for this region. As shown in Figure 1, when the Gabor method could no longer perform ridge recovery on an area, this area was considered to be a distorted area, which was to be segmented as the background. There were two main methods for label generation. The first method was segmentation using the gradient-based threshold algorithm [21], and the second one was manual segmentation using labelme software. The gradient-based segmentation method required high image quality, and not every fingerprint could be well segmented, even if the parameters were tuned.

### 2.2. Data Preprocessing

The RoIAlign module in Mask Region-CNN (RCNN) can handle the misalignment problem while quantizing the region of interest (RoI) coordinates in feature maps by using bilinear interpolation on fixed point values [22]. However, it warps the RoI feature maps into a square size before feeding to the upsampling step. This can lead to further misalignment and information loss when reshaping the ROI feature map back to original size in image coordinates. The nearest method is used in this paper to fill the pixels to get a specific size, aiming to avoid the loss of pixel-wise information when warping regions.

One pixel-wise method was used to adjust the intensity value of each pixel to a same scale as Equation (1), where I (x, y) is the intensity value at pixel (x, y) in input image I, m and v are the image mean and variance, and m0 and v0 are the desired mean and variance after the normalization.

(1) $$x=\{\begin{array}{cc} m_{0}+\sqrt{\frac{{\left(I\left(x,y\right)-m\right)}^{2},v_{0}}{v}}, & l\left(x,y\right)>m\\ m_{0}-\sqrt{\frac{{\left(I\left(x,y\right)-m\right)}^{2},v_{0}}{v}}, & otherwise \end{array}$$

**Figure 2:**
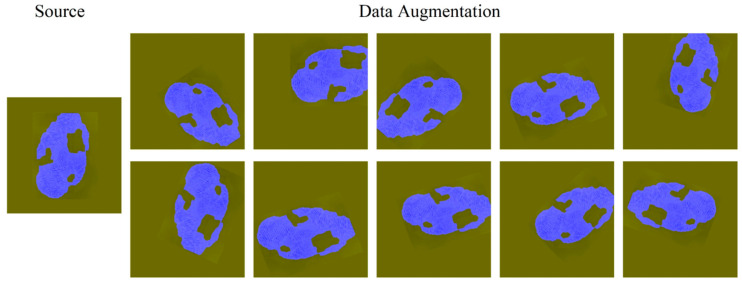
Original fingerprint image and data augmentation.

Data augmentation is essential to teach the network the desired invariance and robustness properties when only few training samples are available. In case of fingerprint images, we primarily need shift and rotation invariance as well as robustness of deformations, gray value variations, and different resolution. ImageDataGenerator in Keras is used to enhance the fingerprint original image, with a rotation range of 180, with a shift range of 0.2, a height shift range of 0.2, a shear range of 0.05, a zoom range of 0.1, a horizontal flip to true, a vertical flip to true, and a fill mode to use nearest. The resulting picture is shown in Figure 2. The blue area is the fingerprint label, which is merged with the fingerprint image. It can also be seen from Figure 2 that the corresponding label of each fingerprint image after the transformation is also equivalently transformed.

This method is for fingerprint images with a resolution of 512 × 512, thus it needs to be cropped and filled. If the original image is larger than 512 × 512, it will be cropped, and for smaller images, the fill will use a grayscale intensity of $m_{0}+\sqrt{({\left(I\left(x,y\right)-m\right)}^{2},v_{0})/v}$. Table 1 shows the original fingerprint image in the three fingerprint databases and the results of resizing and normalization.

### 2.3. Overall Model Architecture

**Figure 3:**
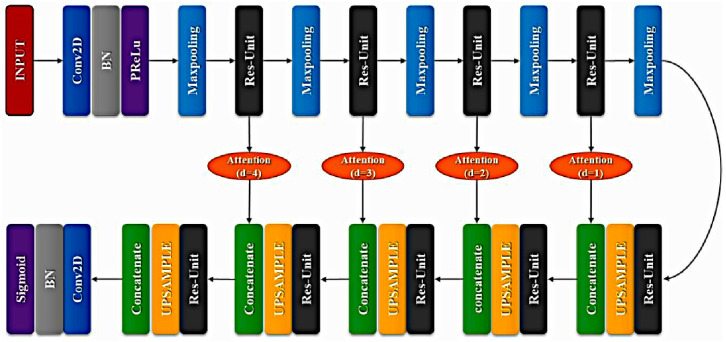
XFinger-Net architecture.

In light of residual attention learning [10] and U-Net [23], we tried to use a combined method to study the segmentation of defect fingerprints. The method used in this paper was constructed by module overlays of multiple attention mechanisms and using residuals. The overall model of the network model is shown in Figure 3. Five pooling layers and five upsampling layers are used in the overall architecture. The use of the attention gate is the main reason why this method is more effective than the original U-Net.

In Figure 3, the depth of the attention gate increases from right to left. The last convolutional layer of the entire network is the sigmoid layer, which outputs the final probability map of 512 × 512 × 1. The layers and the corresponding output sizes in the network architecture are shown in Table 2.

#### 2.3.1. U-Net Based

The method is based on U-Net network. U-Net is a full convolutional network that requires only a small number of training images to work and produces more accurate segmentation. The main idea of a full convolutional network is to supplement the usual shrinking network by successive layers, where the pool operator is replaced by an upsampling operator. Therefore, these layers increase the resolution of the output. The upsampling part of U-net also has a large number of feature channels, which allows the network to propagate context information to higher resolution layers. As a consequence, the expansive path is more or less symmetric to the contracting path and yields a u-shaped architecture. The network does not have any fully connected layers and only uses the valid part of each convolution. U-Net consists of an encoder and a decoder symmetrically on the two sides of the architecture. The contextual information is propagated by the encoder within the rich skip connections, which enables the extraction of hierarchical features with more complexity.

#### 2.3.2. Residual Mechanism

**Figure 4:**
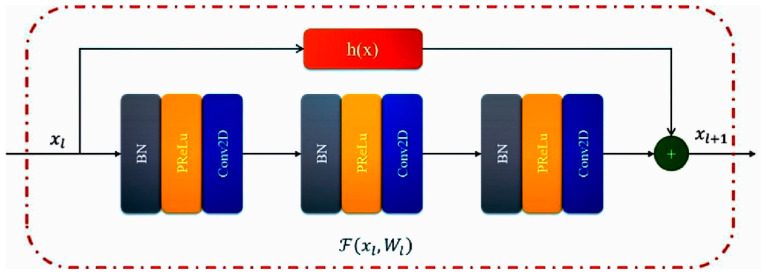
Residual unit used in this paper.

He et al. [24] proposed a residual neural network to facilitate training and address the degradation problem. The residual neural network consists of a series of stacked residual units. Each residual unit can be illustrated as a general form:

(2) $$\begin{array}{l} y_{l}=h\left(x_{l}\right)+F\left(x_{l},W_{l}\right)\\ x_{l+1}=f\left(y_{l}\right) \end{array},$$

where $x_{l}$ and $x_{l+1}$ are the input and the output of the l-th residual unit. F is the residual function, $f\left(y_{l+1}\right)$ is activation function, and $h\left(x_{l}\right)$ is identity mapping function of skip connection. It should be noted that the method used in this study does not use the activation function after the add operation. In [24], h(x) is generally x, and we used a layer of 1×1 convolution; Figure 4 shows the residual unit used in this paper.

#### 2.3.3. Attention Gates in XFinger-Net

Attention gates are commonly used in natural image analysis, knowledge graphs, and language processing (NLP) for image captioning, machine translation, and classification tasks. Trainable attention is categorized as hard-attention and soft-attention. Soft-attention is probabilistic and utilizes standard back-propagation. For instance, soft attention is more recently applied to image classification. The same objective can be achieved by integrating attention gates in a standard CNN model. This does not require the training of multiple models and a large number of extra model parameters. Attention gates progressively suppress feature responses in irrelevant background regions without the requirement to crop a ROI between networks. In [25], a mechanism of attention learning is proposed, but this attention learning is based on the dimension level of 1D. If you use the method of Flatten to reshape the image matrix into a one-dimensional tensor, it may lead to the loss of the important spatial context, which is not preferable. One study [23] proposed a more general attention mechanism:

(3) $$\begin{array}{l} c_{i}^{s}=\Psi \sigma_{1}\left(W_{f}f_{i}^{S}+W_{g}g+b_{g}\right)+b_{\psi } \end{array}$$

Here, $c_{i}^{s}$ represents the compatibility scores at the spatial location i of n total spatial locations in the layer. s represents the given convolutional layer, s∈{1,⋯,S}. $\Psi \in R^{C_{int}}$, is a trainable parameter, $W_{f}\in R^{C_{int}\times C},\;W_{g}\in R^{C_{int}\times C_{g}}$ and bias terms $b_{\psi }\in R,\;b_{g}\in R^{C_{int}}$, $\sigma_{1}$. is a nonlinearity chosen to be PReLU. $f_{i}^{S}$ represents the pixel-wise feature vector. $W_{f}$ and $\left\{\Psi ,b_{\psi }\right\}$ are implemented as 1 × 1 convolution layer, and $\left\{W_{g},b_{g}\right\}$ is a fully connected layer. Once compatibility scores are computed, the normalized attention coefficient $\alpha_{i}^{s}$ is obtained by using the sigmoid unit as follows:

(4) $$\begin{array}{l} \alpha_{i}^{s}=\sigma_{2}\left(c_{i}^{s}\right) \end{array},$$

(5) $$\begin{array}{l} \sigma_{2}\left(x\right)=1/\left(1+exp\left(-x\right)\right) \end{array}.$$

Attention coefficients $\alpha_{i}\in \left[0,\;1\right]$ identify salient image regions, amplify their influence, and suppress irrelevant information in background regions. The output of AGs is the element-wise multiplication of input feature-maps and attention coefficients: ${\hat{f}}_{i}^{s}=f_{i}^{s}\cdot \alpha_{i}^{s}$.

**Figure 5:**
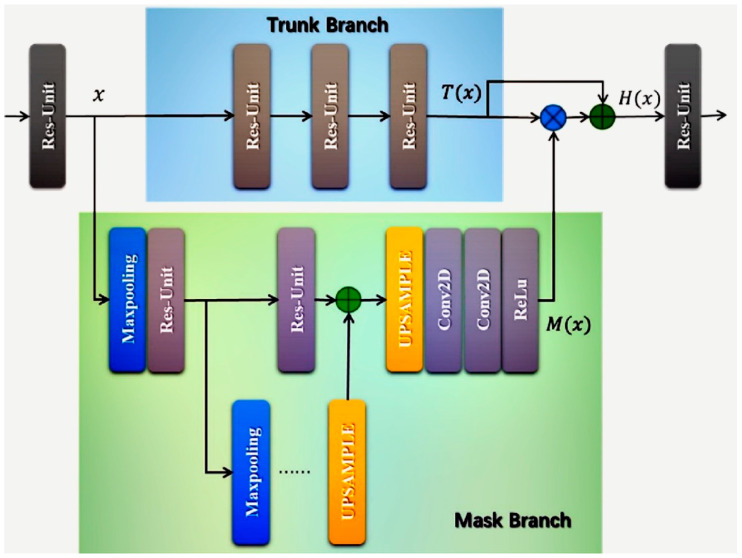
Attention gate architecture. The gradient from the background region decreases in weight when traveling backwards.

In XFinger-Net model, incorporation of the attention gates method makes the foreground area of the fingerprint in the obtained fingerprint image more noticeable. The noise or the like of the background area is almost completely removed from the interference of the fingerprint image feature extraction. The attention gates method is performed before concatenation, except for the associated activation. The attention gate used in XFinger-Net combines the residual mechanism to ensure that the correct features filtered by the attention gate are preserved. Similar to the residual, the attention gate consists of two branches, as shown in Figure 5. The trunk branch performs feature processing, and the mask branch work as feature selectors, which enhance good features and suppress noises from trunk features. Output H of Attention Module as Equation (6), where x is the characteristic tensor of the input, T(x) is the output of the trunk branch, and M(x) is the output of the mask branch. Because of the use of skip connection, attention gate can keep good properties of original features:

(6) $$\begin{array}{l} H\left(x\right)=\left(1+M\left(x\right)\right)\times T\left(x\right) \end{array}$$

#### 2.3.4. Loss Function

The loss function is used to estimate the degree of inconsistency between the predicted value f(x) of the model and the true value Y. The smaller the loss function is, the better the robustness of the model will be. The network predictions, which consist of two volumes having the same resolution as the original input data, are processed through a soft-max layer, which outputs the probability of each voxel to belong to foreground and to background. Several previous approaches resorted to loss functions based on sample re-weighting where foreground regions are given more importance than background ones during learning. In this work, it is not uncommon that the anatomy of interest occupies only a very small region of the scan. This often causes the learning process to get trapped in local minima of the loss function yielding a network whose predictions are strongly biased towards background. As a result, the foreground region is often missing or only partially detected [26]. To solve this problem, a loss function *L* based on the Dice coefficient was proposed. 

The loss $L_{M}$ is defined as Equation (7):

(7) $$L_{M}=1-\left(2\overset{N}{\underset{i=1}{\sum }}s_{i}g_{i}\right)/(\overset{N}{\underset{i=1}{\sum }}s_{i}{}^{2}+\overset{N}{\underset{i=1}{\sum }}g_{i}^{2}),$$

where N is the number of pixels, and $s_{i}$ and $g_{i}$ belong to the binary segmentation and the binary ground truth pixels sets, respectively. The problem studied in this paper is essentially a two-class problem. Therefore, in most cases using the U-Net network model, the cross-entropy of the two-class is often employed. To solve the problem of imbalance between positive and negative samples, cross entropy is redefined as:

(8) $$L_{C}=-\frac{1}{ROI}\underset{ROI}{\sum }\overset{N}{\underset{i=1}{\sum }}(\varepsilon^{+}g_{i}log\left(s_{i}\right)+\varepsilon^{-}\left(1-g_{i}\right)log\left(1-s_{i}\right)),$$

where ROI is the number of pixels in the region of interest, and *ε* is the weight coefficient determined by whether it is a fingerprint area. We use the weighted method to revise the loss function, i.e.,

(9) $$\begin{array}{l} L=\varepsilon_{1}L_{c}+\varepsilon_{2}L_{M} \end{array},$$

where $\varepsilon_{1}$ is the weight of the loss function $L_{c},$ and $\varepsilon_{2}$ is the weight of the loss function $L_{M}$.

#### 2.3.5. Implementation Details

The architecture of this method was constructed using the Keras and the TensorFlow [27] libraries. The network parameters used a random initialization method, and the backward propagation of the network is based on Adam. In the Adam optimizer, we set the initial values of Learning Rate (LR) to 0.001, $\beta_{1}=0.9$, $\beta_{1}=0.995$. In addition, this method uses a variable learning rate strategy, using the ReduceLROnPlateau function in Keras, with a factor of 0.1 and a patience of 15, indicating if the domain went to plateau after 15 epoches, the learning rate would be reduced to 0.1 times the original. The training of all the models was performed with an NVIDIA 1060 GPU. Therefore, the batch size was limited during the fitting process, and the training data were 8:2 compared with the verification data. In terms of loss correction, we used a proportion of 6:4, that is, 40% of the weight was the cross-entropy of the two classification, and 60% of the weight was Direct Current (DC) loss.

## 3. Experiments and Results

### 3.1. Performance Metrics

We evaluated the performance of the learner in this paper. The fingerprint segmentation is a two-category task that we could measure with accuracy, precision, recall, Fl score and Area Under Curve (AUC). Finally, the Precision-Recall (PR) curve and the Receiver Operating Characteristic (ROC) curve were used to analyze the results

### 3.2. Train of XFinger-Net and ResU-net

**Figure 6:**
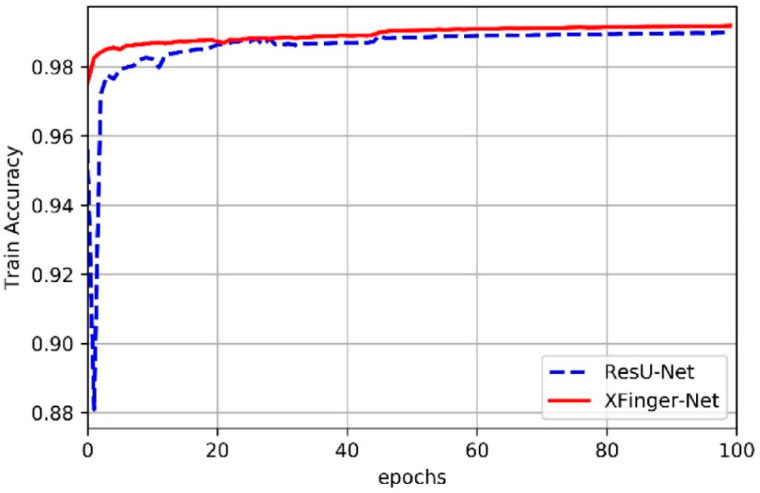
Train accuracy of XFinger-Net and ResU-net with epoch during training.

**Figure 7:**
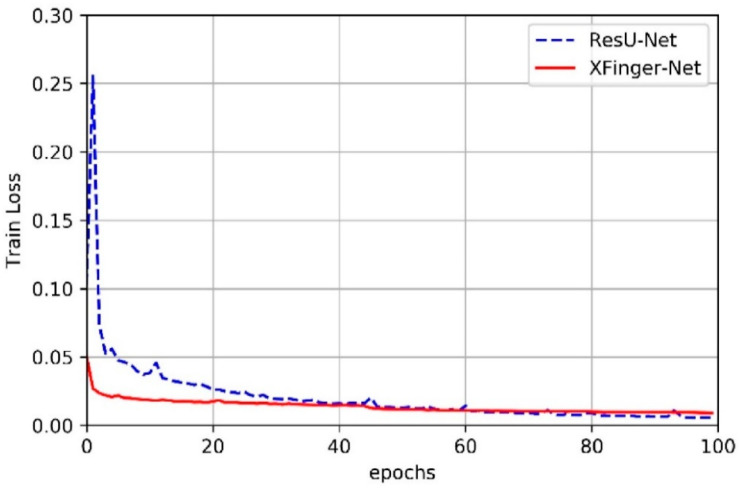
Train loss of XFinger-Net and ResU-net with epoch during training.

**Figure 8:**
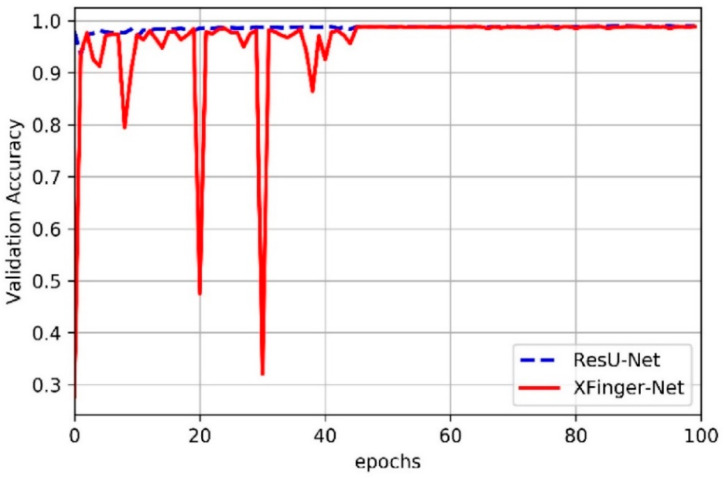
Validation accuracy of XFinger-Net and ResU-net with epoch during training.

**Figure 9:**
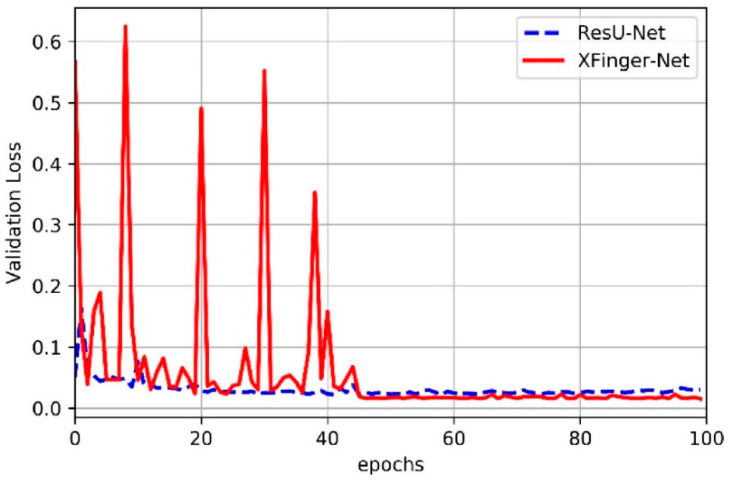
Validation loss of XFinger-Net and ResU-net with epoch during training.

In this article, we did not slice the fingerprint image but used the fingerprint normalized image with the resolution of 512 × 512 as input of XFinger-Net and ResU-net [28]. Figure 6 shows the trend of train accuracy of XFinger-Net and ResU-net with epoch during training. Figure 7 shows the trend of train loss of XFinger-Net and ResU-net with epoch during training. Figure 8 shows the trend of validation accuracy of XFinger-Net and ResU-net with epoch during training. Figure 9 shows the trend of validation loss of XFinger-Net and ResU-net with epoch during training.

It can be seen from Figure 6, Figure 7, Figure 8 and Figure 9 that accuracy and loss remained constant in the 50th generation, and there was not much obvious fluctuation in the subsequent training. The training accuracy of XFinger-Net finally stabilized at 0.9919, and the training accuracy of ResU-Net finally stabilized at 0.9889. The training loss of XFinger-Net finally stabilized at 0.0092, and the training loss of ResU-Net finally stabilized at 0.0056. The loss of XFinger-Net was larger than that of ResU-Net, because XFinger-Net and ResU-Net did not use the same loss function. ResU-Net still used the two-class cross entropy as the loss. The validation accuracy of XFinger-Net finally stabilized at 0.9891, and the validation accuracy of ResU-Net finally stabilized at0.9900. The validation loss of XFinger-Net finally stabilized at 0.0153, and the validation loss of ResU-Net finally stabilized at 0.0300.

We used the training dataset for model input to get the forward inference results of the training set including the validation set, which was later used to compare the test set inference results. Part of the results are shown in Table 3. Since the original image below is in the training set and verification set, it can be seen that the predicted result is basically the same as the ground truth. It can be said to be completely consistent with the complete fingerprint, but there is still a slight pixel-level difference from the defective fingerprint.

### 3.3. Test Results and Comparison of XFinger-Net, ResU-Net, Gradient-Based Methods

The test dataset we used was described above and was obtained from the fingerprint dataset acquired by Liver20 fingerprint collector, FVC2000, FVC2002, FVC2004, FVC2006, and the LivDet dataset. There are 160 pictures in the test set, which were cropped to a size of 512 × 512 and normalized. Some test results of XFinger-Net are demonstrated in Table 4.

**Figure 10:**
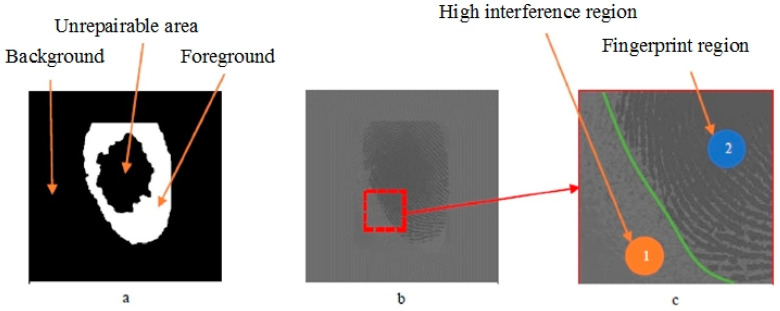
Fingerprint segmentation with high noise background. (**a**) The segmentation results of the XFinger-Net method; (**b**) extended and normalized fingerprint images; (**c**) fingerprint block details.

Through Table 4, it can be seen that the method is almost perfect for the segmentation of a complete fingerprint. It has excellent effects for most of the defective fingerprints. In Figure 10, the area marked as 1 is the background area, and that marked as 2 is the foreground area. There is a fingerprint-like afterimage, but the segmentation results in Figure 10 demonstrate that the method well separates the high interference region.

Threshold segmentation using fingerprint gradients has been the most commonly used fingerprint image segmentation method. The gradient of a pixel is not directly calculated by the Sobel operator but refers to a gradient of a pixel with the surrounding diameter *r*, as Equation (10). The search traversal of the threshold is performed by the Otsu’s method to maximize the interclass variance.

(10) $$\begin{array}{l} \{\begin{array}{c} G_{x}\left(x,y\right)=\left(\overset{\frac{r}{2}}{\underset{u=-\frac{r}{2}}{\sum }}\overset{\frac{r}{2}}{\underset{v=-\frac{r}{2}}{\sum }}G_{x}\left(u,v\right)\right)/r^{2}\\ G_{y}\left(x,y\right)=\left(\overset{\frac{r}{2}}{\underset{u=-\frac{r}{2}}{\sum }}\overset{\frac{r}{2}}{\underset{v=-\frac{r}{2}}{\sum }}G_{y}\left(u,v\right)\right)/r^{2} \end{array} \end{array}$$

The U-Net method used in this paper is not the original network architecture in [23], but the ResU-Net architecture; the biggest difference between them is that the latter includes a residual mechanism. FingerNet [29] is a method proposed by Tang for fingerprint feature point extraction, in which the segmentation result is a by-product and assists the extraction of feature points. Multi-scale Atrous Spatial Pyramid Pooling (ASPP) method is used to generate fingerprint images with 8 × 8. The block is the fingerprint segmentation map of the basic unit, but it only extracts the fingerprint foreground and does not segment the unrecoverable area in the foreground. The most classical segmentation method is based on the grayscale intensity variance of the fingerprint image, which also uses a fingerprint image block of 8 × 8 as the basic unit.

**Figure 11:**
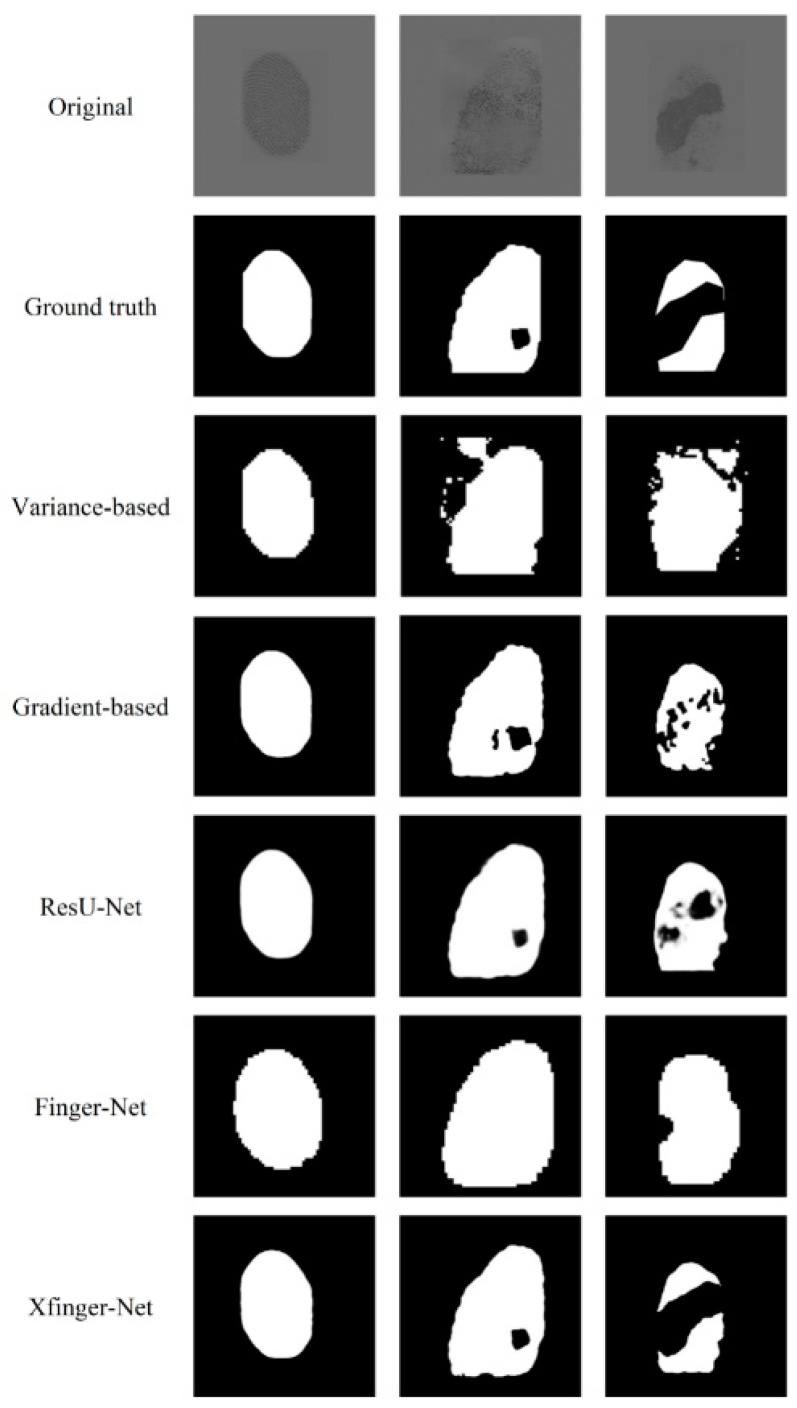
Comparison of the effects of XFinger-Net, ResU-Net, gradient-based methods.

The segmentation effects of different segmentation methods are compared and shown in Figure 11. The method based on gradient and gray intensity variance has obvious weaknesses in that their segmentation effect largely depends on the quality of the fingerprint images. As shown in the comparison of the gradient-based column in Figure 11, the fingerprint in the first row is obviously of good quality, thus the segmentation effect is better, but the fingerprint of poor quality in the second and the third rows has a large deviation in segmentation. The gradient-based method has an accuracy of 0.88, and we no longer use any performance metrics comparing with XFinger-Net and ResU-Net. The goal of FingerNet is to extract the foreground of the fingerprint without segmenting the defect area in the foreground. It can be seen to have a good recognition effect on the edge area of the fingerprint.

**Figure 12:**
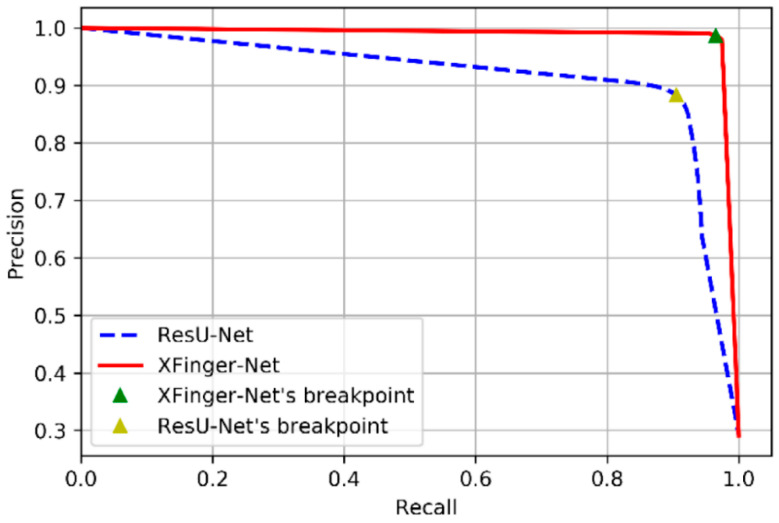
PR curve of XFinger-Net and ResU-Net.

**Figure 13:**
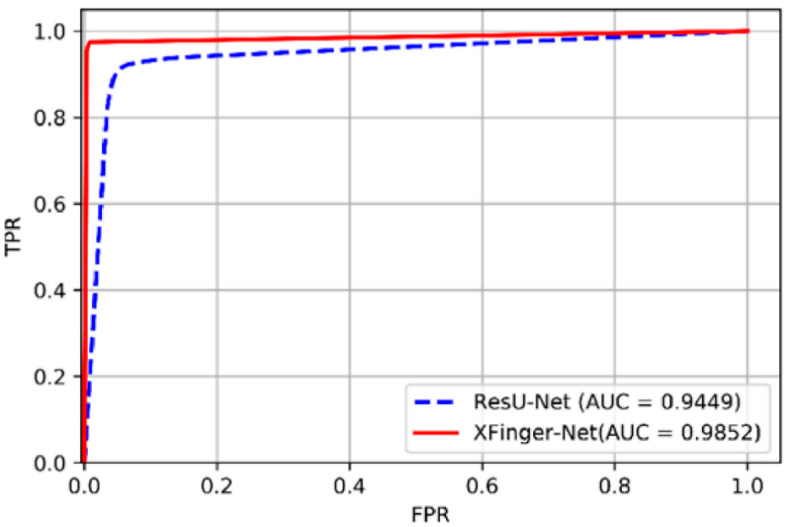
ROC curve of XFinger-Net and ResU-Net.

The PR curves of XFinger-Net and ResU-Net are shown in Figure 12, and their ROC curves are presented in Figure 13. The performances of XFinger-Net and ResU-Net in the test set ire shown in Table 5, where accuracy refers to the ratio of the number of samples correctly predicted to the total number of samples; recall refers to the ratio of the number of samples predicted to be positive to the number of true positive samples; specificity refers to the ratio of the number of samples predicted to be negative to the number of true negative samples; precision refers to the probability that the sample cpredicted to be a positive example is indeed a positive example; AUC (area under Curve) is defined as the area enclosed by the ROC curve and the coordinate axis (the closer the AUC is to 1.0, the higher the authenticity of the segmentation methods is); the dice coefficient is used to measure the similarity of two sets, whose value range is from 0 to 1 (the closer to 1, the better the segmentation methods; and F1 score is defined as the harmonic mean of precision and recall, as shown in Equation (11).

(11) $$F_{1}=2\cdot \left(Precision\cdot Recall\right)/\left(Precision+Recall\right)$$

The break-event point (BEP) is the value when the precision is equal to the recall rate. The BEP of XFinger-Net figure was 0.9863, and that of ResU-Net figure was 0.8831. The performance indicators of XFinger-Net were all around 0.98, indicating that the defect fingerprint had a fairly good segmentation effect. It can be seen from Table 5 that XFinger-Net’s performance was better than ResU-Net’s.

**Figure 14:**
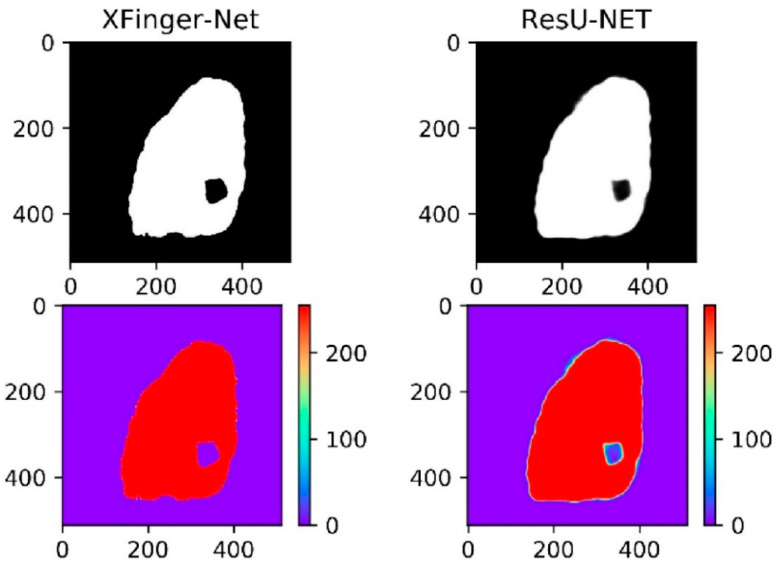
Thermal map of the segmentation result of XFinger-Net and ResU-Net.

**Figure 15:**
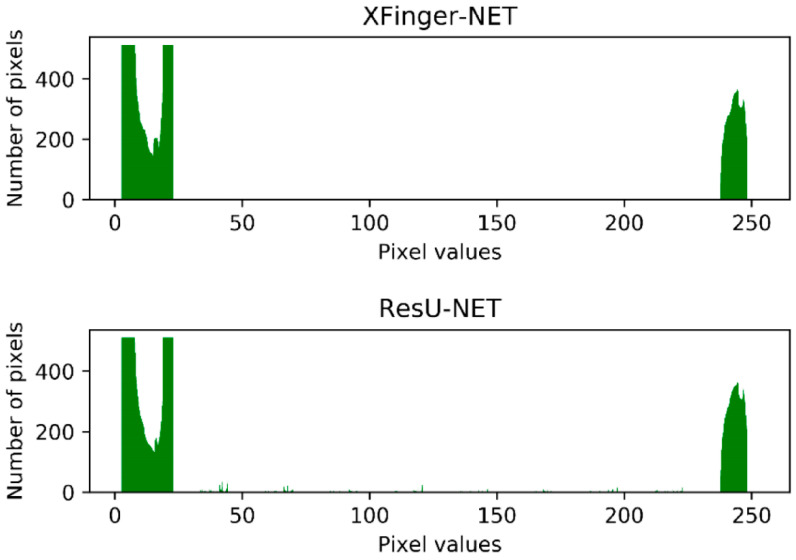
Histogram of the segmentation result of XFinger-Net and ResU-Net.

In fact, in Figure 11, the ResU-Net segmentation diagram seems vague, which is not an illusion. Among the three methods, the gradient-based segmentation method obtained a 1-bit image, and the ResU-Net and the segmented image obtained by the method were 8-bit images of one channel, that is, the pixel value q ∈ (0, 255). It can be seen from Figure 14 that the foreground color of the ResU-Net segmentation was neither close to red nor to purple and had a large number of intermediate pixel values. As shown in Figure 15, the pixel distribution of the segmentation map obtained by XFinger-net was polarized, focusing on 0 and 255, and the pixel distribution of some pixels in the segmentation graph obtained by ResU-Net was between 0 and 255. That means that the classification threshold of the segmentation result graph obtained by ResU-Net was not clear enough, which resulted in blurring of the foreground edge of the fingerprint in the result image, leading to a decrease in accuracy.

## 4. Conclusions

This paper proposes the XFinger-net method for segmenting PDFIs. The method is completely different from the traditional segmentation methods based on artificially defined fingerprint features. It uses the deep learning method for segmentation of partially defective and high noise fingerprints, avoiding the use of multiple and complex filtering and other preprocessing processes. In the network structure, XFinger-Net is similar to U-shaped nesting. In the attention gate, its mask branch is also a U-like structure. If an attention gate is understood as a feature filter, the use of multiple attention gates ensures that multiple features will be used as the basis for segmentation and gives high weight to the location of the region of interest. According to the method, the loss function is corrected by weighting DC and binary classification cross entropy. This method is proposed for dealing with fingerprint images with high noise and defects. It presented strong robustness in the final test, which not only suppressed the background noise but also effectively identified the noise and the pollution of the foreground area of the fingerprint. Compared with the traditional gradient-based fingerprint segmentation [28], the method was nearly 10% more accurate. We compared it with ResU-Net using multiple evaluation methods such as accuracy and dice coefficient. XFinger-Net showed better performance with high accuracy and precision, including the segmented fingerprint foreground boundary. The fingerprint segmentation method in this paper is promising, but this method also has deviation in the segmentation for some defective fingerprint. Therefore, an appropriate increase in network depth can be considered in future research, such as the use of other network architectures in the trunk branch of attention gates instead of a single series residual unit. It is important to emphasize that global features are very important for segmentation of defective fingerprints, thus it is recommended to have complete fingerprint images as input.

Moreover, in recent years, finger vein recognition has become more attractive due to some obvious advantages [30], such as: in-vivo recognition, high anti-counterfeiting, high acceptability, high stability, etc. However, for some finger vein images, its vein structure is too simple, and the useful information is too little; traditional recognition methods usually perform poorly on such images. Therefore, we consider applying the segmentation algorithm proposed in this paper to finger vein recognition in the future to realize the potential of the presented methodology.

## Figures and Tables

**Table 1:** Original fingerprint image and normalized image from three datasets.

FVC	LiveDet	Liver20
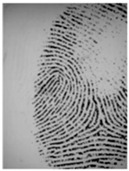	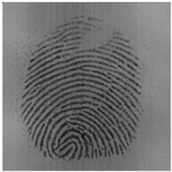	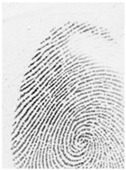
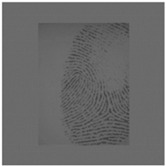	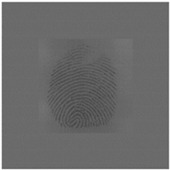	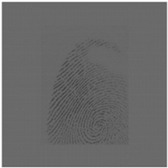

**Table 2:** Optimal results for two different load cases.

Layer	Outsize	Layer	Outsize	Layer	Outsize
INPUT	512 × 512 × 1			SIGMOID	512 × 512 × 1
CONV1	512 × 512 × 32			CONV2	512 × 512 × 32
MAXPOOL1	256 × 256 × 32			UPSAMPLE5	512 × 512 × 32
RES-UNIT1	256 × 256 × 32	AG1	256 × 256 × 32	RES-UNIT9	256 × 256 × 32
MAXPOOL2	128 × 128 × 32			UPSAMPLE4	128 × 128 × 64
RES-UNIT2	128 × 128 × 64	AG2	128 × 128 × 64	RES-UNIT8	128 × 128 × 64
MAXPOOL3	64 × 64 × 64			UPSAMPLE3	128 × 128 × 128
RES-UNIT3	64 × 64 × 128	AG3	64 × 64 × 128	RES-UNIT7	64 × 64 × 128
MAXPOOL4	32 × 32 × 128			UPSAMPLE2	64 × 64 × 256
RES-UNIT4	32 × 32 × 256	AG4	32 × 32 × 256	RES-UNIT6	32 × 32 × 256
MAXPOOL5	16 × 16 × 256			UPSAMPLE1	32 × 32 × 512
RES-UNIT5	16 × 16 × 512				

**Table 3:** Forward reasoning results of training set and validate set.

Original	Truth	Predict	Original	Truth	Predict
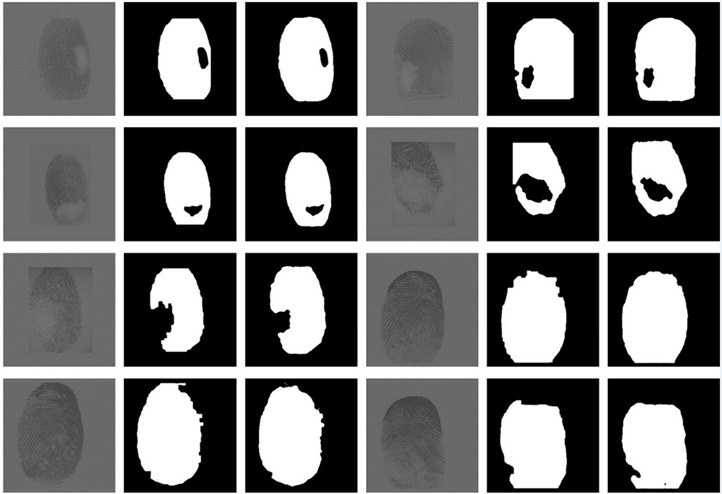					

**Table 4:** Forward reasoning results of test set.

Original	Truth	Predict	Original	Truth	Predict
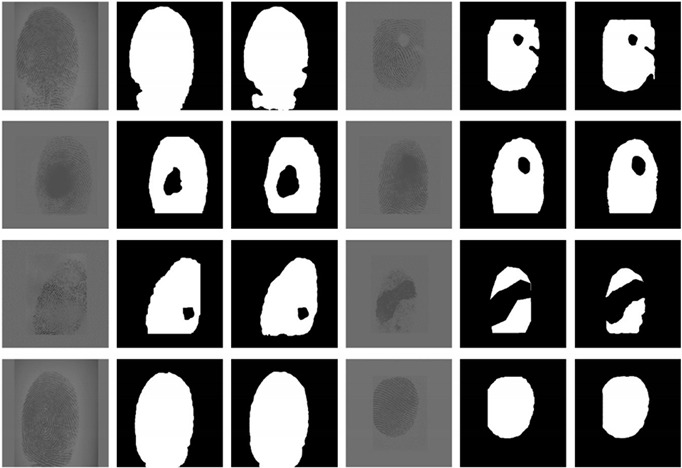					

**Table 5:** The score of the segmentation algorithm on test dataset.

Method	Accuracy	Recall	Specificity	Precision	AUC	F1 Score	Dice Coefficient
XFinger-Net	0.9859	0.9649	0.9945	0.9863	0.9852	0.9755	0.9841
ResU-Net	0.9371	0.9050	0.9504	0.8831	0.9449	0.8939	0.8921
FingerNet	0.9287	/	/	/	/	/	/
Gradient-Based	0.8816	/	/	/	/	/	/
Variance-Based	0.8351	/	/	/	/	/	/

AUC: area under the curve.
